# Supplementary material for: Premature polyadenylation of MAGI3 produces a dominantly-acting oncogene in human breast cancer
Source: eLife. 2016 May 20;5:e14730. doi: 10.7554/eLife.14730 (PMC4905742; doi:10.7554/eLife.14730)
Supplement: Supplementary file 1. — DOI: http://dx.doi.org/10.7554/eLife.14730.016 [file elife-14730-supp1.docx]

**Supplementary File 1. Comprehensive list of candidate MAGI3-interacting proteins in MCF10A cells.**

| **Total Peptides** | **Gene Symbol** |  | **Total Peptides** | **Gene Symbol** |
| --- | --- | --- | --- | --- |
| 112 | YAP1 |  | 21 | ARHGAP1 |
| 85 | SERPINB5 |  | 21 | BZW1 |
| 79 | AHNAK2 |  | 21 | CUL5 |
| 58 | PLIN3 |  | 21 | RAP1GDS1 |
| 53 | DIAPH1 |  | 21 | TNKS1BP1 |
| 48 | TJP2 |  | 21 | VASP |
| 43 | ASS1 |  | 20 | ARHGEF1 |
| 43 | EHD2 |  | 20 | DAK |
| 42 | SRP54 |  | 20 | ECHDC1 |
| 40 | EHD1 |  | 20 | NDRG1 |
| 40 | MVP |  | 20 | PPP1R13L |
| 37 | FAM114A2 |  | 20 | STXBP2 |
| 36 | STIP1 |  | 19 | KIAA1033 |
| 33 | PPL |  | 19 | PTPN11 |
| 32 | FRYL |  | 19 | STXBP4 |
| 32 | SUCLG2 |  | 19 | SYAP1 |
| 30 | FKBP5 |  | 19 | WWC1 |
| 30 | KIAA0196 |  | 18 | ARHGEF16 |
| 30 | UBQLN1 |  | 18 | CCDC6 |
| 29 | ACADM |  | 18 | COQ9 |
| 29 | TSNAX |  | 18 | MAP2K1 |
| 27 | ATP6V1E1 |  | 18 | NSFL1C |
| 27 | FAM21A |  | 18 | RIC8A |
| 27 | PAK2 |  | 18 | SH3GLB1 |
| 27 | PSME1 |  | 17 | ACY1 |
| 27 | PSME2 |  | 17 | CLINT1 |
| 26 | PPP1R7 |  | 17 | CSE1L |
| 25 | CNDP2 |  | 17 | EEA1 |
| 25 | CUL1 |  | 17 | EFHD2 |
| 24 | EHD4 |  | 17 | FUBP1 |
| 24 | LRBA |  | 17 | SLK |
| 24 | PDXDC1 |  | 17 | SUCLA2 |
| 23 | ANXA8 |  | 16 | EPS8L2 |
| 23 | ARFGAP2 |  | 16 | FKBP15 |
| 23 | DCTN2 |  | 16 | KLC1 |
| 23 | MAGI1 |  | 16 | RABGGTA |
| 22 | FAM129B |  | 16 | SSH3 |
