# Supplementary material for: Premature polyadenylation of MAGI3 produces a dominantly-acting oncogene in human breast cancer
Source: eLife. 2016 May 20;5:e14730. doi: 10.7554/eLife.14730 (PMC4905742; doi:10.7554/eLife.14730)
Supplement: Supplementary file 2. — DOI: http://dx.doi.org/10.7554/eLife.14730.017 [file elife-14730-supp2.docx]

**Supplementary File 2. Top Human PDZ Domains Predicted to Bind the YAP PDZ-Binding Motif.**

| **Rank** | **Gene Symbol** | **Domain** |
| --- | --- | --- |
| 1 | MAGI3 | PDZ6 |
| 2 | MAST3 | PDZ1 |
| 3 | PDZK1 | PDZ3 |
| 4 | MPDZ | PDZ2 |
| 5 | SLC9A3R1 | PDZ2 |
| 6 | SHANK3 | PDZ1 |
| 7 | MAGI1 | PDZ6 |
| 8 | SHANK2 | PDZ1 |
| 9 | INADL | PDZ10 |
